# A non-healing corneal ulcer as the presenting feature of type 1 diabetes mellitus: a case report

**DOI:** 10.1186/1752-1947-5-539

**Published:** 2011-11-04

**Authors:** Alexander S Ioannidis, Sofia L Zagora, Alfred W Wechsler

**Affiliations:** 1 Sydney Eye Hospital, 8 Macquarie Street, Sydney, NSW 2000, Australia

## Abstract

**Introduction:**

Diabetic keratopathy is a rare complication of diabetes mellitus. This case illustrates the importance of checking blood sugar levels of patients with non-healing corneal ulcers to rule out the possibility of undiagnosed diabetes mellitus.

**Case presentation:**

We report the unusual case of a 24-year-old southeast Asian woman who presented with a sterile corneal ulcer to our hospital and later was found to be diabetic after a prolonged hospital stay. Despite all efforts, the corneal ulcer had failed to heal until treatment for previously undiagnosed diabetes was started. The sterile corneal ulcer began to heal once blood sugar levels began to normalize.

**Conclusions:**

Diabetic keratopathy is a rare complication of diabetes mellitus and needs to be considered as a diagnosis in younger patients with non-healing sterile corneal ulcers. Blood sugar levels should be checked in these cases for undiagnosed diabetes mellitus.

## Introduction

We report an unusual case of a 24-year-old southeast Asian woman who presented with a sterile corneal ulcer to our hospital and later was found to be diabetic. Her corneal ulcer had failed to heal until her blood sugar levels began to normalize. Diabetic keratopathy is a rare complication of diabetes mellitus and needs to be considered as a diagnosis in younger patients with non-healing sterile corneal ulcers. In a previous publication, a 44-year-old man presented in a similar fashion, although in that instance the condition was bilateral [1]. The case in our report highlights the importance of investigating patients who present with unexplained corneal ulceration to exclude undiagnosed diabetes mellitus.

## Case presentation

**Figure 1:**
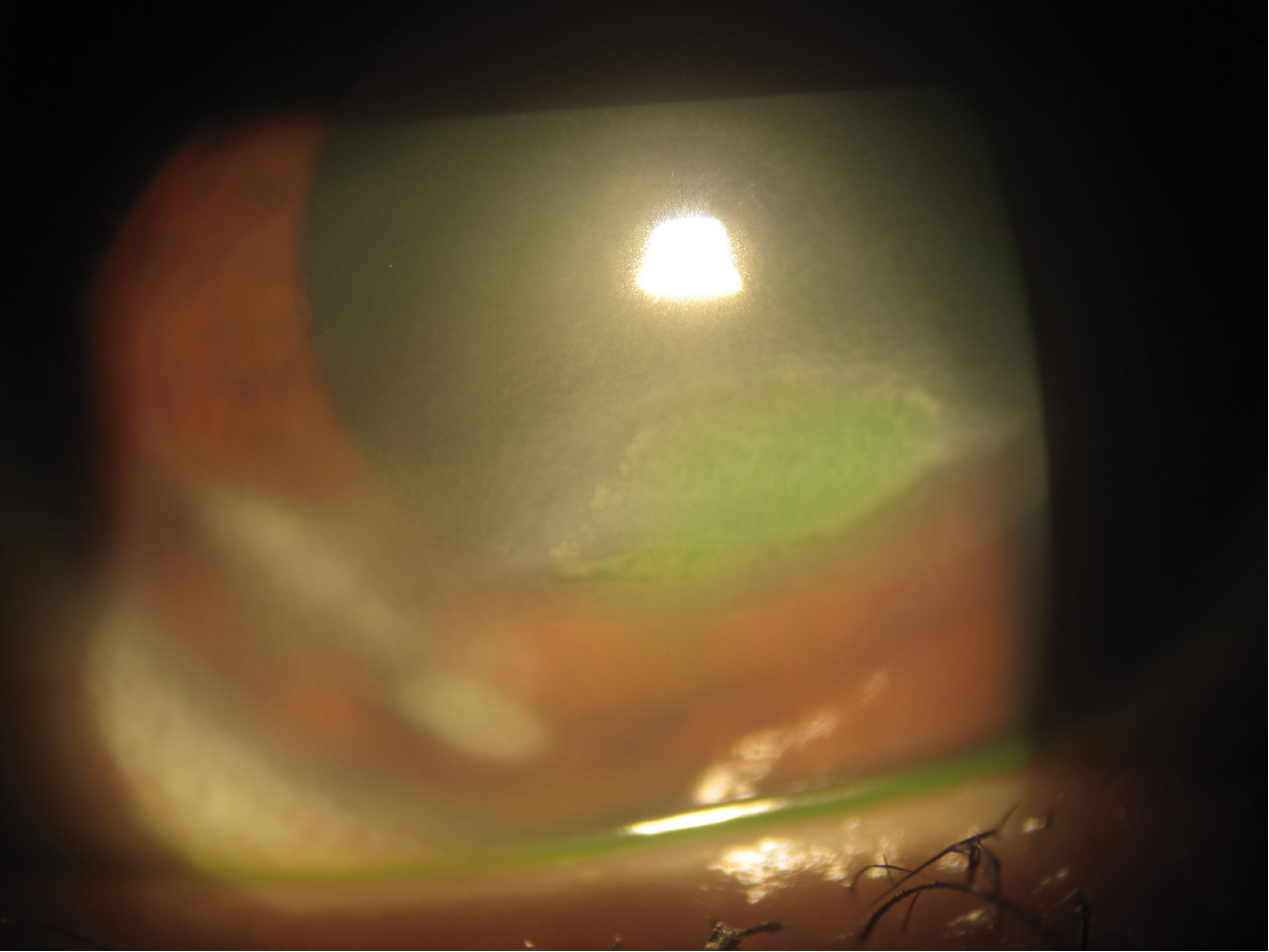
**Color photograph of the right eye shows the ulcer and an area of paracentral intrastromal scarring**.

A 24-year-old southeast Asian woman was admitted with a history of a white spot on the right cornea and increasing discomfort. On examination, her vision was 6/36 on the right and 6/9 on the left. She had a corneal ulcer measuring 5.5 × 2 mm on her right cornea. A small localized area of scarring was present lateral to where the defect was present (Figure 1). There was a +1 cell reaction in her right anterior chamber. She had a history of bilateral anterior uveitis. Corneal sensation was normal in both eyes. There were early bilateral posterior subcapsular cataracts.

**Figure 2:**
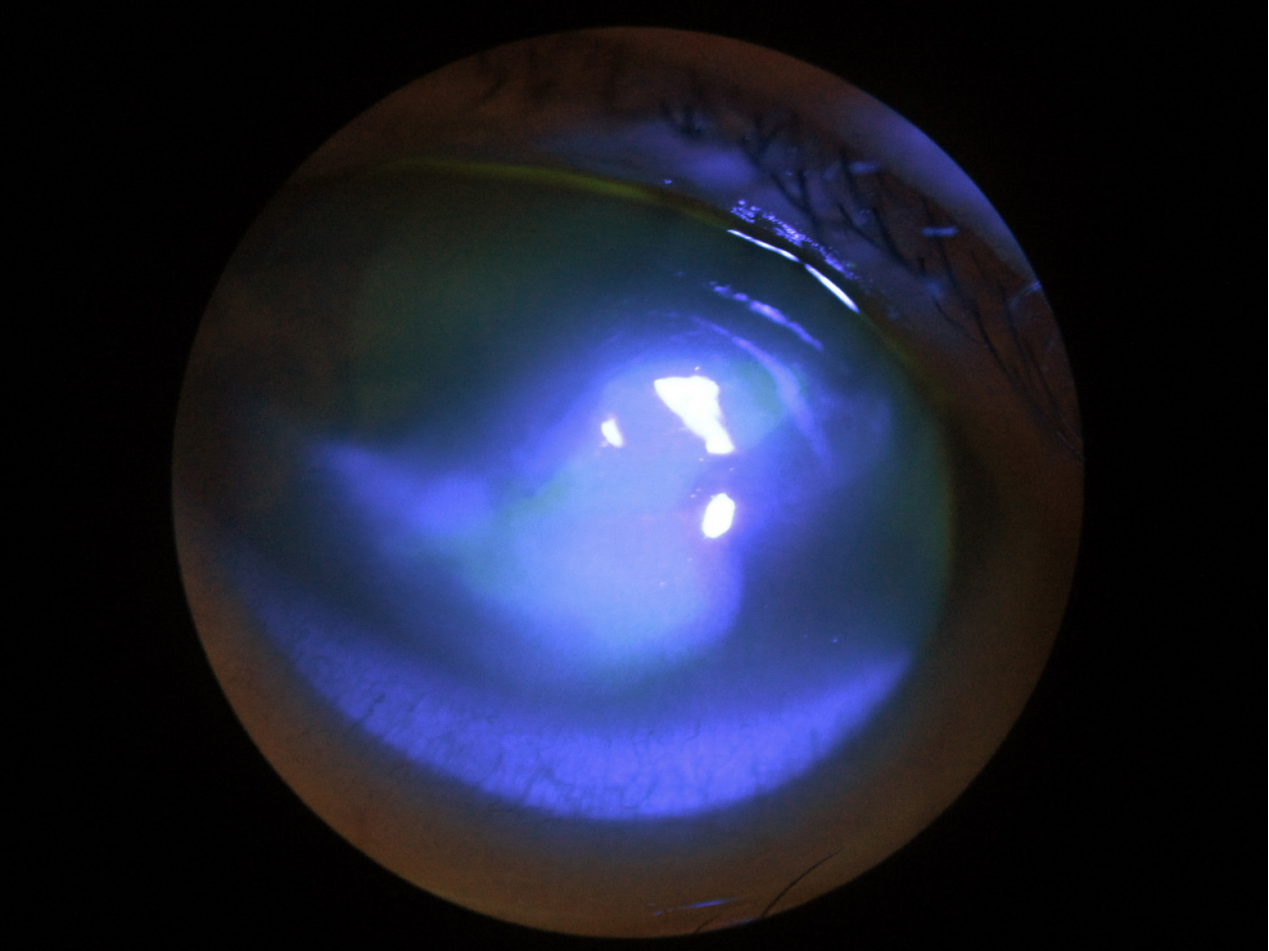
**Cobalt blue photograph of the right eye**. Fluorescein dye was used to highlight the large central ulcer (green stain).

In view of the findings, corneal scrapes were taken for microscopy, culture, and sensitivity. Virology assays inclusive of herpes simplex virus and varicella-zoster virus polymerase chain reaction were performed. Our patient had normal C-reactive protein, rheumatoid factor, anti-nuclear antibody, extractable nuclear antigen, syphilis, and hepatitis B and C serology. She was started on topical g. cephalothin 5% and g. gentamicin 0.9% hourly for 48 hours. She made a mild initial improvement and was changed to topical g. chloramphenicol 1% four times each day and g. prednisolone 0.5% four times each day once her microbiology and virology results were negative. A bandage contact lens was inserted to facilitate healing (Figure 2).

In the third week of admission, she complained of a headache and was found to be mildly tachycardic. She was apyrexial with no reported malaise. A urinary dipstick analysis was performed, and her urinary glucose level was 21 mmol/L. Blood glucose was urgently requested and was found to be 23 mmol/L. A blood gas analysis showed a pH of 7.38, a partial pressure of carbon dioxide (pCO_2_) of 44.7 mmHg, and a partial pressure of oxygen (pO_2_) of 89.5 mmHg.

**Figure 3:**
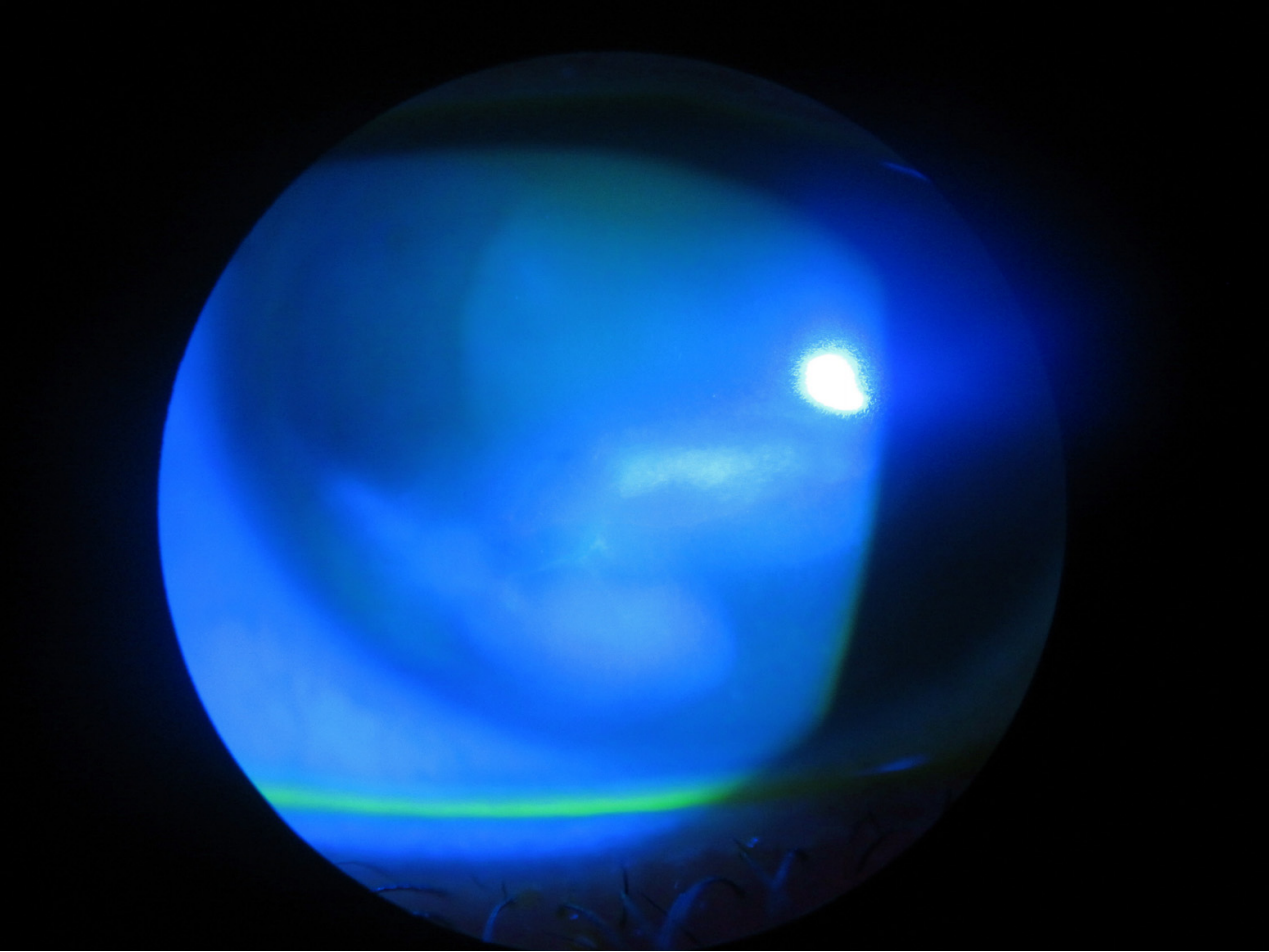
**Cobalt blue photograph of the right eye shows a smaller ulcer (green stain)**. The ulcer began to heal rapidly once insulin treatment was initiated.

She was transferred to the care of the medical team and a diagnosis of type 1 diabetes was made. She was started on treatment with insulin. Her corneal ulcer persisted and punctal plugs were inserted to increase the tear film and facilitate healing. Autologous serum drops were started every two hours during waking hours. There was a rapid reduction of the epithelial defect as her blood glucose levels normalized (Figure 3).

**Figure 4:**
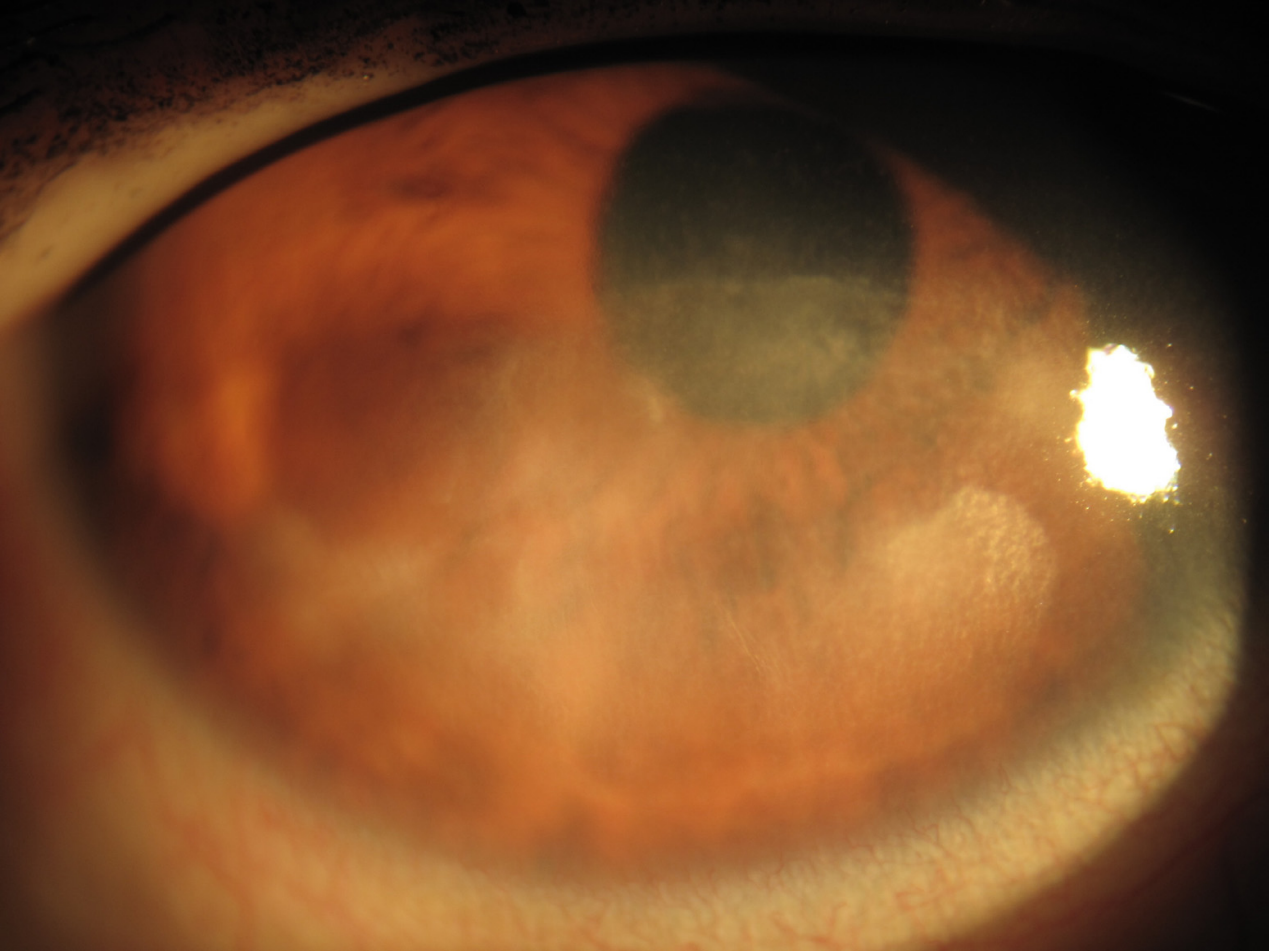
**Color photograph of the right eye at one month**. The defect has healed, leaving a diffuse area of scarring.

Four days after insulin treatment was started, her ulcer had healed and she was discharged from the hospital and follow-up was conducted at her local diabetes clinic. At a one-month review in the eye clinic, her ulcer remained healed, leaving a localized area of subepithelial scarring (Figure 4).

## Discussion

The ocular features of diabetes mellitus have been described in other reports. Impaired glucose metabolism typically results in a localized microangiopathy that affects primarily the retinal vasculature and that produces the classic lesions in the fundus with microaneurysms, intraretinal hemorrhages, exudation, and new vessel formation [2]. A combination of good glycemic control and regular visits to the eye clinic can often slow or halt the progression of the disease.

Diabetic keratopathy is a rare complication of the condition. In this setting, impaired epithelial healing is thought to be a consequence of an abnormal aldose reductase pathway and secondary accumulation of polyol within the epithelial and endothelial cells and thus result in cellular dysfunction [3,4]. This results in delayed healing responses and loss of epithelial adhesion to the basement membrane, increasing the risk of recurrent corneal erosions. Minor trauma and ocular manipulation with contact lenses can also produce chronic non-healing defects [5]. Our patient had no history of trauma or contact lens use. Other ocular features of diabetes mellitus include reduced corneal sensation and tear production and basement membrane thickening [5-7].

It is important when considering the diagnosis of diabetic keratopathy to exclude other treatable causes of non-healing defects, such as anterior basement membrane dystrophy and recurrent erosion syndrome. Treatment options in cases of a persistent ulceration include the use of frequent lubrication, bandage contact lenses, topical autologous serum drops, and patching. If conservative measures fail, it may be necessary to perform temporary tarsorrhaphy to facilitate healing.

Other conditions that delay epithelial healing need to be identified and treated accordingly. Hence, coexisting neurotrophic keratopathy needs to be excluded by a careful assessment of corneal sensation. Dry eye disease can also delay healing and can be identified with rose-bengal staining of the cornea and conjunctiva and use of the Schirmer test. Nocturnal lagophthalmos needs to be managed with appropriate nocturnal padding and lubrication.

Animal studies have shown that the opioid antagonist naltrexone and insulin used topically can facilitate healing in diabetic rats by enhancing DNA synthesis and re-epithelialization through alterations in local opioid growth factors [8,9]. In the future, these agents may be approved for use in the treatment of diabetic keratopathy.

## Conclusions

Four days after insulin treatment was started, our patient's ulcer had healed and she was discharged from the hospital and follow-up was arranged at her local diabetes clinic. The diagnosis of diabetes mellitus was thus made somewhat incidentally. Our patient did not report weight loss, polyuria, or polydipsia to facilitate the diagnosis of diabetes, and her only complaint was a brief history of headaches while in the hospital. Her urinary glucose level was checked as part of an investigation for transient tachycardia and elevated glucose levels were detected.

Using a combination of a bandage contact lens, temporary punctal plugs, and autologous serum drops proved useful in facilitating ulcer healing. Although the corneal ulcer showed initial signs of healing, it was after the blood sugar levels began to normalize that the ulcer fully healed.

## Consent

Written informed consent was obtained from the patient for publication of this case report and any accompanying images. A copy of the written consent is available for review by the Editor-in-Chief of this journal.

## Competing interests

The authors declare that they have no competing interests.

## Authors' contributions

ASI analyzed and interpreted the patient data regarding the clinical presentation. SLZ and AWW were major contributors in writing the manuscript. All authors read and approved the final manuscript.
